# Right-Sided Congenital Diaphragmatic Hernia With Gut Malrotation: A Rare Case Report

**DOI:** 10.7759/cureus.65602

**Published:** 2024-07-28

**Authors:** Neha Thorbole, Sudhir Malwade, Abhishek Denge, Shivani Kale

**Affiliations:** 1 Department of Paediatrics, Dr. D.Y. Patil Medical College, Hospital and Research Centre, Dr. D.Y. Patil Vidyapeeth (Deemed to be University), Pune, IND

**Keywords:** bochdalek's hernia, congential diaphragmatic hernia, duodenal atresia, exploratory laparotomy, pulmonary hypertension, pulmonary hypoplasia, cdh

## Abstract

Congenital diaphragmatic hernia (CDH) is a rare surgical cause of respiratory distress in neonates. CDH is caused by the protrusion of the abdominal contents into the thoracic cavity due to the failure of the pleuroperitoneal canal to close by eight weeks of gestation. We present the case of a full-term, female child, weighing 2.85 kg at birth, born by normal vaginal delivery to a 21-year-old primigravida admitted at our level III neonatal intensive care unit (NICU). Antenatal obstetric ultrasonography suggested duodenal atresia. After birth, the child was found to have right-sided CDH with gut malrotation. Intraoperative laparotomy revealed a right Bochdalek posterolateral defect with herniation of small bowel loops and a portion of the right lobe of the liver into the chest cavity and minimally malrotated cecum in the right iliac fossa (RIF). This case highlights the critical need for early detection and multidisciplinary management of congenital anomalies. Effective management requires a multidisciplinary approach, including prenatal counseling, careful surgical intervention, and intensive neonatal care to optimize respiratory and cardiovascular outcomes for affected infants.

## Introduction

Congenital diaphragmatic hernia (CDH) is the abnormality of the diaphragm that allows the contents of the abdomen to protrude into the thoracic cavity, putting neonates at significant risk for cardiac and pulmonary problems. The defect could be a small aperture in the margin of the posterior muscle or the diaphragm's total absence [1]. It occurs in approximately one out of every 2,000 to 4,000 live births [2]. The incidence is more common in males compared to females [3]. The location of the defect determines the classification of CDH: Bochdalek hernias, or posterior-lateral hernias, account for 70-75% of hernias; anterior defects, or Morgagni hernias, account for 23-28%; and uncommon central hernia accounts for 2-7%. Right-sided hernias are less common in Bochdalek hernias, which occur on the left side of the body 85% of the time and the right side 13% of the time [4]. CDH can occur as an isolated condition or in association with other anomalies. One common associated condition is intestinal malrotation, which is seen in up to 45% of CDH cases [5]. Intestinal malrotation is a congenital anatomical anomaly resulting from abnormal rotation of the gut as it returns to the abdominal cavity during embryogenesis [6]. One in every 6,000 live newborns is thought to have mid-gut malrotation [7]. CDH is commonly associated with intestinal malrotation due to the abnormal prenatal positioning of the intestines caused by herniation into the chest cavity during early fetal life. This herniation disrupts the normal rotation and fixation process of the intestines, leading to malrotation [7,8]. The main factors affecting the prognosis of CDH patients include pulmonary hypoplasia, pulmonary hypertension, and related abnormalities (cardiac defects, chromosomal abnormalities, or other structural malformations) [9]. Pulmonary hypoplasia and pulmonary hypertension are indeed the major causes of morbidity and mortality in patients with CDH [10]. Here, we discuss the case of right-sided CDH with gut malrotation in a female child.

## Case presentation

A full-term female newborn, weighing 2.85 kg at birth, was born via normal vaginal delivery to a 21-year-old primigravida at a gestational age of 39 weeks and three days at a tertiary care center in western India. She was an unregistered case. An antenatal obstetric ultrasound performed at 35 weeks and one day of gestation suggested duodenal atresia. An anomaly scan was not done. The baby did not require resuscitation at birth. The DOWNES score was zero, and the child was on room air and did not require any respiratory support, with Apgar scores of 7/10 at one minute and 9/10 at five minutes. She was shifted to the neonatal intensive care unit (NICU) for further evaluation of antenatal findings.

**Figure 1:**
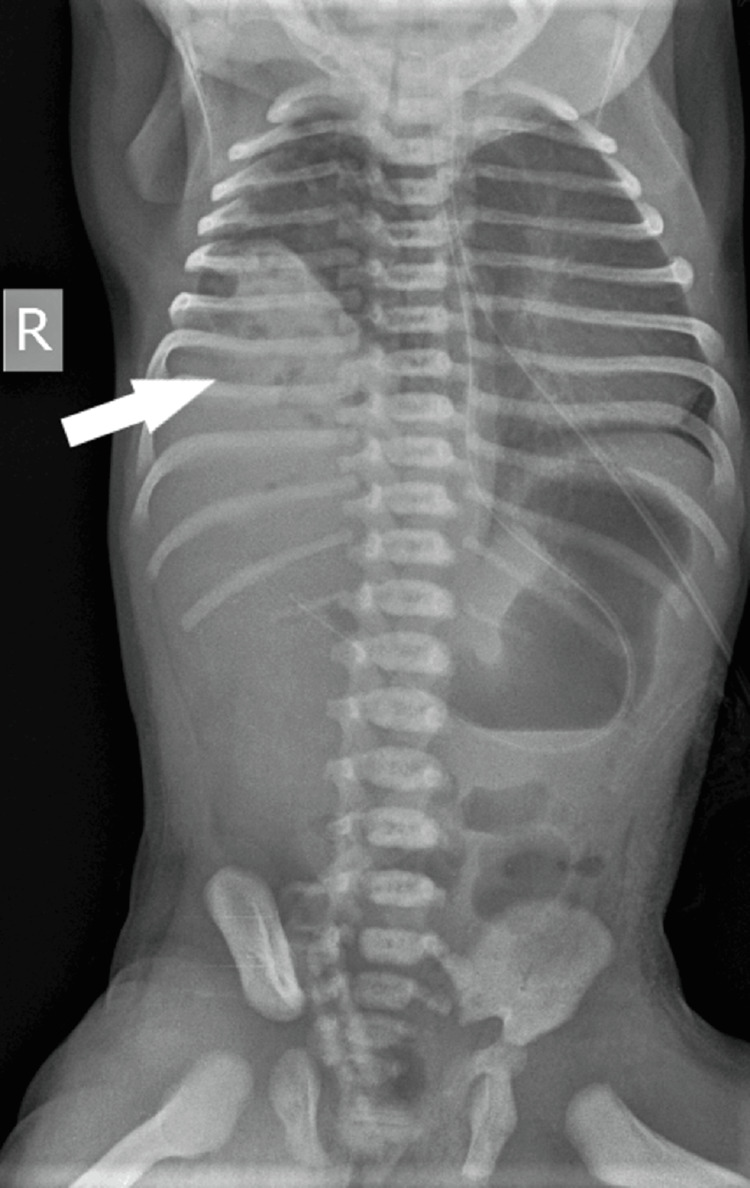
X-ray showing herniation of abdominal contents in the right hemithorax (arrow).

**Figure 2:**
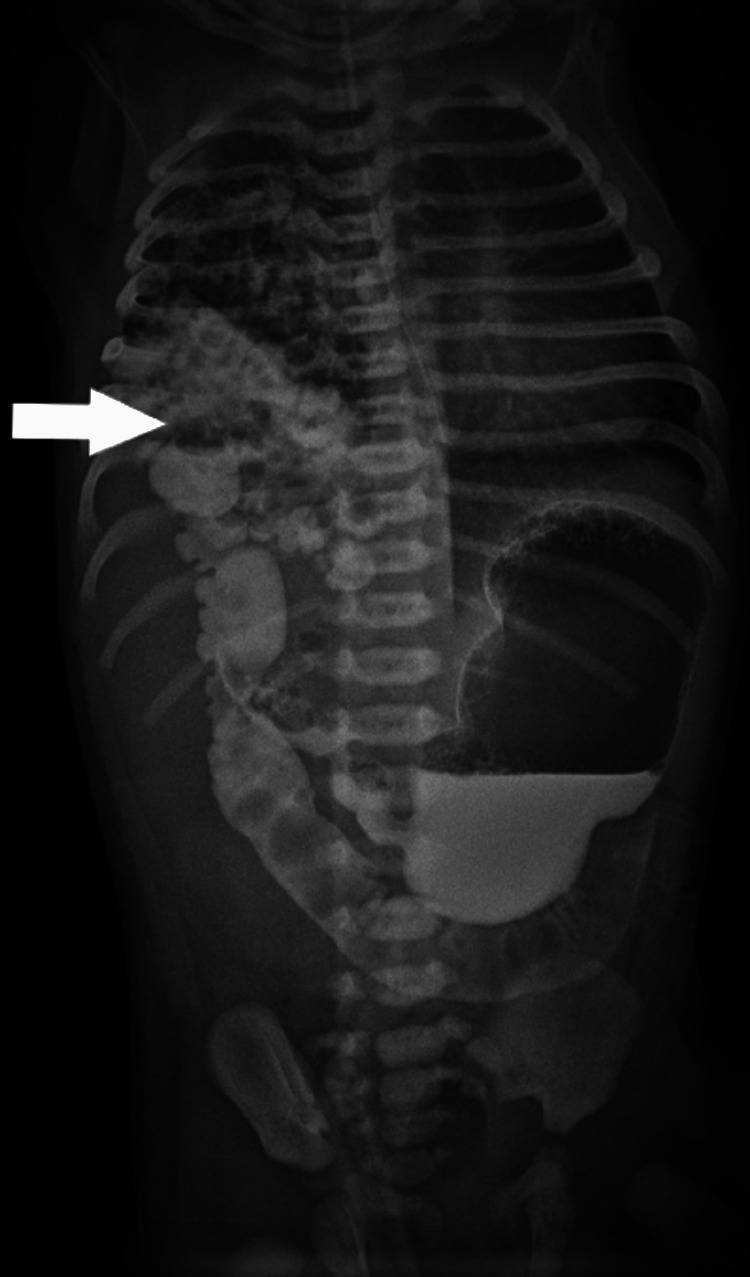
X-ray showing protrusion of bowel in the right hemithorax (arrow) along with malrotation of the gut.

In the NICU, the child had greenish aspirate, for which she was kept nil by mouth (NBM) and started on intravenous fluids. An X-ray was done along with routine investigations. The X-ray showed herniation of bowel loops in the right hemithorax (Figure 1). A pediatric surgeon advised a dye study to be done at 24 hours of life. The dye study showed loops of bowel in the right hemithorax, suggestive of right-sided CDH along with a reversed superior mesenteric artery (SMA)/superior mesenteric vein (SMV) relationship (Figure 2). We ruled out vertebral defects, anal atresia, cardiac defect, tracheoesophageal fistula, renal anomalies, and limb abnormalities (VACTERL) association by performing an X-ray of the whole spine to look for any vertebral anomaly. Clinical examination did not reveal any anorectal malformation. A 2D echocardiography was normal. A nasogastric tube (NGT) could be easily passed, ruling out tracheoesophageal fistula. An ultrasound was done to rule out renal anomalies, and an infantogram showed no limb abnormalities.

**Figure 3:**
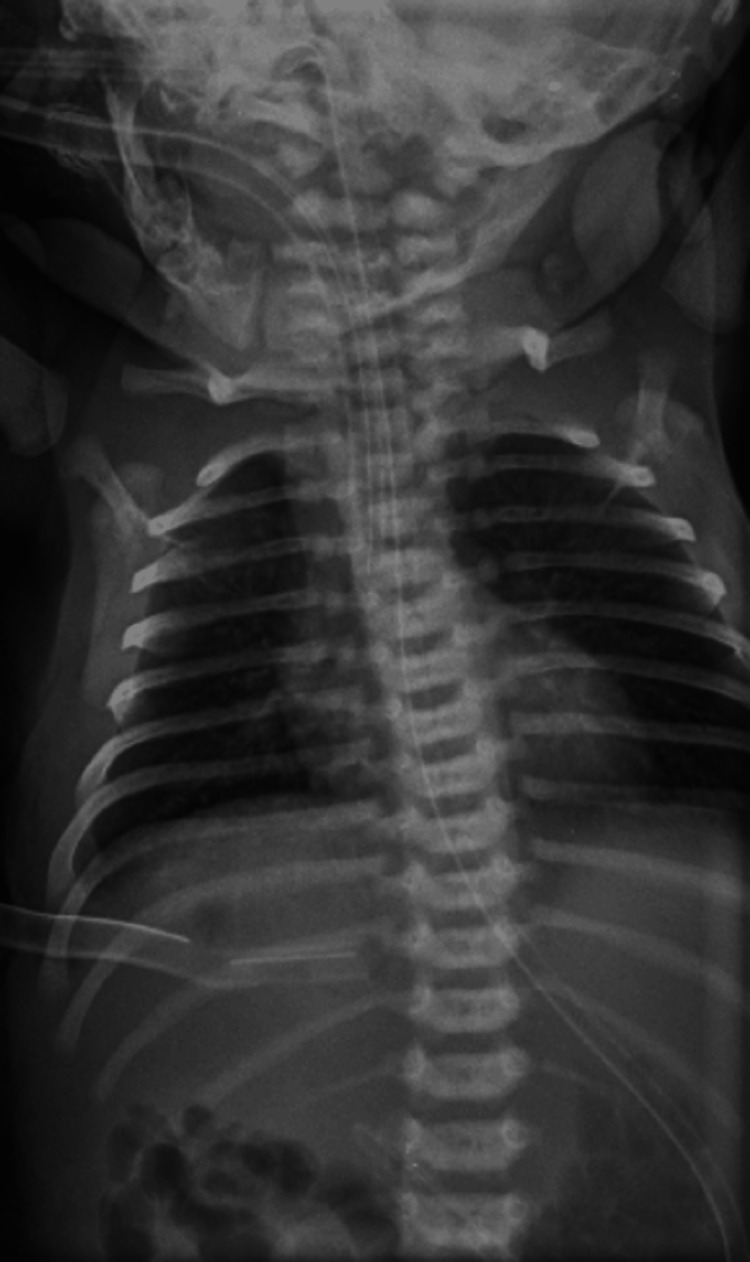
X-ray showing complete repair of the CDH. CDH: congenital diaphragmatic hernia.

As the child was hemodynamically stable, she was taken for surgical management on day 2 of life. An exploratory laparotomy was performed. Intraoperative findings revealed a right Bochdalek posterolateral defect with herniation of small bowel loops and a portion of the right lobe of the liver into the thoracic cavity and a minimally malrotated cecum in the right iliac fossa (RIF). The distal jejunum was in midline. The herniated bowel loops were reduced along with the right lobe of the liver, and a diaphragmatic defect was repaired (Figure 3). There was no evidence of duodenal atresia intraoperatively. Surgery was completed in three and a half hours; intraoperative blood loss was 10 ml. Postoperatively, the child was shifted to the NICU, intubated, and was on dobutamine support at 10 mcg/kg/min, which was gradually tapered and stopped. After 48 hours, the child was extubated and started on minimal feeds. After two days, she was on full enteral feeds on day 7 of life and was stable on room air. She was discharged home on day 12 of life.

## Discussion

CDH is one of the most important surgical causes of respiratory distress in neonates. The combination of CDH and gut malrotation, as observed in our case, underscores the need for a high index of suspicion and comprehensive evaluation in neonates presenting with respiratory distress and gastrointestinal symptoms.

The development of the diaphragm begins around the fourth week of gestation. It involves the fusion of several embryonic structures, including the septum transversum and the pleuroperitoneal membranes [11]. CDH is caused by the protrusion of the abdominal contents into the thorax due to the failure of the pleuroperitoneal canal to close by eight weeks of gestation. The midgut herniates through the umbilical cord in the fourth or fifth week of pregnancy and re-enters the abdominal cavity in the ninth or tenth week. During the ninth to tenth week of gestation, as the midgut returns to the abdominal cavity, it undergoes a 270° anticlockwise rotation around the superior mesenteric artery. This rotation is crucial for the formation of the duodenal arch and the correct placement of the intestines within the abdominal cavity. When there is a CDH, the displacement of abdominal viscera into the thoracic cavity can distort normal intestinal anatomy and disrupt the proper fixation of the intestines. This displacement and distortion are significant factors contributing to complications such as intestinal malrotation and potential bowel obstruction in neonates with CDH [12].

CDH is associated with significant respiratory distress and pulmonary hypertension in neonates, primarily due to pulmonary hypoplasia [13]. Persistent pulmonary hypertension (PPH) after successful repair of CDH is indeed a significant risk factor for morbidity and mortality in infants [14].

In our case, the child was stable on room air and had no pulmonary hypertension. The absence of additional anomalies improved the overall prognosis and allowed for focused management of the identified conditions. The surgical intervention was timely and crucial for the neonate's survival. The successful reduction of the herniated organs and repair of the diaphragmatic defect underscore the importance of prompt surgical management in improving outcomes for infants with CDH. The optimal timing for surgical treatment is controversial, but there is consensus in the literature that surgery should be postponed until the newborn is medically and physiologically stable.

In this case report, the antenatal ultrasound done at 35 weeks indicated duodenal atresia but could not identify the CDH, possibly due to the herniation of bowel loops occurring near-term gestation, which in typical cases is diagnosed between 24 and 26 weeks of gestation on antenatal ultrasound [15]. This case emphasizes the critical need for early detection and multidisciplinary management of congenital anomalies. Comprehensive prenatal care, including anomaly scans and timely ultrasound evaluations, can significantly influence perinatal outcomes. Furthermore, the case underscores the importance of a coordinated approach involving obstetricians, neonatologists, pediatric surgeons, and intensive care specialists to optimize the management and prognosis of neonates with complex congenital conditions such as CDH and malrotation. This case also highlights the gaps in prenatal care that need to be addressed to improve maternal and neonatal health outcomes.

## Conclusions

CDH presents significant challenges due to its association with pulmonary hypoplasia and subsequent respiratory distress. Despite advancements in prenatal diagnosis and surgical techniques, CDH remains a complex condition with high morbidity and mortality risks, particularly due to complications such as persistent pulmonary hypertension of the newborn (PPHN) post-repair. Effective management requires a multidisciplinary approach, including prenatal counseling, careful surgical intervention, and intensive neonatal care, to optimize respiratory and cardiovascular outcomes for affected infants. The successful outcome in this case reflects the importance of early diagnosis, timely surgical intervention, and comprehensive postoperative care in managing neonates with congenital diaphragmatic hernia and associated anomalies. Ongoing research and clinical advancements are crucial for improving the long-term prognosis and quality of life for these patients.
